# Identification of candidate infection genes from the model entomopathogenic nematode *Heterorhabditis bacteriophora*

**DOI:** 10.1186/s12864-016-3468-6

**Published:** 2017-01-03

**Authors:** Jonathan Vadnal, Ramesh Ratnappan, Melissa Keaney, Eric Kenney, Ioannis Eleftherianos, Damien O’Halloran, John M. Hawdon

**Affiliations:** 1 Department of Biological Sciences, George Washington University, Science and Engineering Hall, suite 6000, 800 22nd Street NW, Washington DC, 20052 USA; 2 Department of Microbiology Immunology and Tropical Medicine, George Washington University Medical Center, Washington DC, 20037 USA; 3 Institute for Neuroscience, George Washington University, 636 Ross Hall, 2300 I Street NW, Washington DC, 20052 USA

**Keywords:** *Heterorhabditis bacteriophora*, RNA-seq, Hemolymph, Parasitism, Nematode

## Abstract

**Background:**

Despite important progress in the field of innate immunity, our understanding of host immune responses to parasitic nematode infections lags behind that of responses to microbes. A limiting factor has been the obligate requirement for a vertebrate host which has hindered investigation of the parasitic nematode infective process. The nematode parasite *Heterorhabditis bacteriophora* offers great potential as a model to genetically dissect the process of infection. With its mutualistic *Photorhabdus luminescens* bacteria, *H. bacteriophora* invades multiple species of insects, which it kills and exploits as a food source for the development of several nematode generations. The ability to culture the life cycle of *H. bacteriophora* on plates growing the bacterial symbiont makes it a very exciting model of parasitic infection that can be used to unlock the molecular events occurring during infection of a host that are inaccessible using vertebrate hosts.

**Results:**

To profile the transcriptional response of an infective nematode during the early stage of infection, we performed next generation RNA sequencing on *H. bacteriophora* IJs incubated in *Manduca sexta* hemolymph plasma for 9 h. A subset of up-regulated and down-regulated genes were validated using qRT-PCR. Comparative analysis of the transcriptome with untreated controls found a number of differentially expressed genes (DEGs) which cover a number of different functional categories. A subset of DEGs is conserved across Clade V parasitic nematodes revealing an array of candidate parasitic genes.

**Conclusions:**

Our analysis reveals transcriptional changes in the regulation of a large number of genes, most of which have not been shown previously to play a role in the process of infection. A significant proportion of these genes are unique to parasitic nematodes, suggesting the identification of a group of parasitism factors within nematodes. Future studies using these candidates may provide functional insight into the process of nematode parasitism and also the molecular evolution of parasitism within nematodes.

**Electronic supplementary material:**

The online version of this article (doi:10.1186/s12864-016-3468-6) contains supplementary material, which is available to authorized users.

## Background

Parasitic nematodes (PN) continue to place a considerable burden on human health and agricultural production. PN diseases cause a variety of unfavorable conditions ranging from lethargy to fever, diarrhea, blindness and death. An estimated 1–1.2 billion people are infected with PNs leading to an increase of up to 52.1 million disability adjusted life years with hookworm infections contributing more than 40% of the lost years alone [1]. Current control strategies of deworming, while effective in the short-term, are inadequate due to frequent reinfection and the development of drug resistant nematode populations. Furthermore, attempts at creating a vaccine have been hindered due to the lack of good animal models and effective antigens [2].

While PN infections are understood to be immunomodulatory in nature, the molecular mechanisms of infection are poorly understood [3]. While efforts have been made to study parasitism in PNs directly, the requirement of a vertebrate host for development makes in vitro cultivation currently impossible, thereby making access to parasitic life stages and the interactions between the host immune system and the parasite difficult [4]. Even though a great deal has been learned about nematode biology and development using the free-living nematode *Caenorhabditis elegans*, it is not a parasite and thus does not allow for relevant investigations of PN infection mechanisms. For this reason, interest in developing *Heterorhabditis bacteriophora* into a model organism in order to study nematode parasitism has recently grown [5–8].

*Heterorhabditis bacteriophora* is a member of the *Eurhabditis* clade, which also contains other PNs such as the vertebrate hookworms *Ancylostoma ceylanicum* and *Necator americanus* as well as the model organism *C. elegans. Heterorhabditis bacteriophora* is an entomopathogenic nematode (EPN) which uses host insects and the mutualistic bacteria *Photorhabdus luminescens* to successfully reproduce [9]. The free-living stage of *H. bacteriophora*, the infective juvenile (IJ), is a developmentally arrested stage analogous to the infective stage of hookworms and the dauer of *C. elegans* [10, 11]. The IJs seek host insects to colonize and reproduce. Once established, the IJs resume their development and progress through the complete life cycle of *H. bacteriophora*. After 2 to 3 generations of reproduction, the nutrition of the host’s cadaver is exhausted and juveniles begin to arrest in mass as IJs. These IJs leave the cadaver and begin to search for a new host. Unlike hookworms and most other PNs, *H. bacteriophora* and its bacterial symbiont *P. luminescens* can be manipulated and cultured in vitro. Additionally, advanced molecular tools (e.g. gene silencing by RNAi) are being developed for *H. bacteriophora* as well as the recent publication of its genome, making *H. bacteriophora* potentially an excellent alternative model for nematode parasitism [5, 8, 12–14]. Furthermore, the ability to propagate *H. bacteriophora* in the immunology model *Drosophila melanogaster*, allows the study of host responses to PN infection mechanisms [15–17].

While the basic tools to develop *H. bacteriophora* as a model organism have been or are in the process of being developed [8, 13], genes directly involved in parasitism are still poorly understood. Studies examining the transcriptome of *H. bacteriophora* have been performed, but our results described here is the first study, to our knowledge, to utilize advanced next-generation sequencing technologies and the published *H. bacteriophora* genome to examine the transcriptional program during host invasion [18, 19]. A better understanding of this crucial transition period could help define parasitism genes and possibly allow the establishment of new interventions that prevent infection of hosts. To begin teasing apart the molecular biology of the early infection, we performed next generation RNA sequencing on *H. bacteriophora* IJs incubated in *Manduca sexta* hemolymph plasma for 9 h. Comparative analysis of the transcriptome with untreated controls found a number of differentially expressed genes (DEGs) which cover a number of different functional categories. Furthermore, a subset of the DEGs is conserved across Clade V parasitic nematodes. This subset of genes may serve as potential targets for future studies investigating nematode parasitism.

## Results

### Illumina sequencing

RNA-sequencing using the Illumina HiSeq4000 platform was performed on *H. bacteriophora* (TT01 strain) IJs soaked for 9 h in *M. sexta* hemolymph plasma in order to identify nematode genes that are expressed during the initial stages of insect infection. Total RNA was collected from six independent samples (three 9 h hemolymph plasma treated and three untreated 0 h controls) to characterize the transcriptome and measure differential expression of genes. An outline of the pipeline used to analyze RNA-seq data is shown in Additional file 1: Figure S1. After quality checks and trimming, RNA-sequencing yielded an average of ~41.8 million reads per sample, with an average of 89.1% of control and 81.3% of hemolymph-treated reads mapping to the *H. bacteriophora* genome. There was an average of 4,084,703 and 8,600,584 unmapped reads for control and treated IJ respectively, making the total number of mapped reads 33,554,746 and 37,399,072. Further details of the read mapping are shown in Additional file 1: Table S1. Of the 20,964 genes contained within the *H. bacteriophora* genome, 1641 were significantly (*p* < 0.05) differentially expressed after a 9-h exposure to hemolymph plasma. 881 of these DEGs were upregulated (fold change ≥ 2) and 760 were downregulated (fold change ≤ −2) relative to the 0 h control.

### Validation of RNA-seq by qRT-PCR

**Fig. 1:**
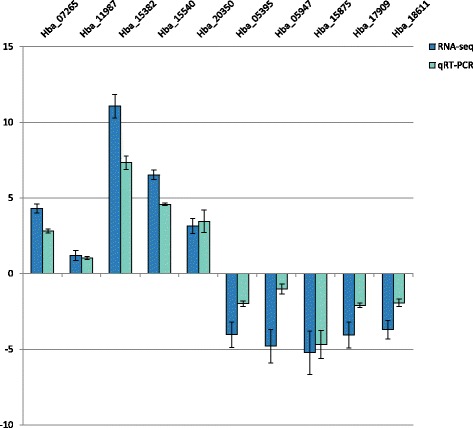
qRT-PCR of genes identified as differentially expressed by RNA-seq. Quantitative RT-PCR using *rpl-32* as an expression control was performed on samples used for RNA-seq to confirm changes in expression. Additionally, qRT-PCR was also performed on a set of samples prepared independently from the samples used for RNA-seq to further validate expression changes. ΔΔC_T_ values are relative to matched control samples. Similar changes in expression were seen in both the RNA-seq and independent samples. *Error bars* represent SEM

In order to confirm changes in expression observed by differential expression analysis of RNA-seq data, qRT-PCR was performed on selected genes. Genes with the largest fold changes, either positive or negative and with significant differential expression (*p* < 0.05), were used for validation. Genes used to test upregulation were Hba_07265 (logFC = 5.08), Hba_11987 (logFC = 3.02), Hba_15382 (logFC = 7.97), Hba_15540 (logFC = 6.47), and Hba_20350 (logFC = 3.75). Hba_05395 (logFC = −3.17), Hba_05947 (logFC = −3.33), Hba_15875 (logFC = −4.44), Hba_17909 (logFC = −3.31), and Hba_18611 (logFC = −2.81) were used as representatives of downregulated genes. In order to confirm differential regulation, qRT-PCR was also performed on untreated control and hemolymph plasma soaked IJs made independently of the samples used for RNA-seq. Using *rpl-32* as an expression control, the directionality of the calculated ΔΔC_T_ values for the treated IJ used for RNA-seq agreed with the changes observed in the RNA-seq differential analysis (Fig. 1). The changes observed by RNA-seq were also present in the samples made exclusively for qRT-PCR.

### GO analysis of RNA-seq data

**Fig. 2:**
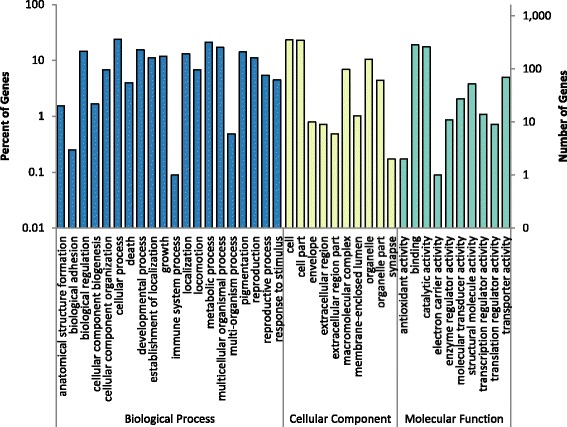
Common GO annotations of DEGs. GO terms were condensed into higher order functional categories using WEGO, in order to more easily understand global changes of expression. Changes were seen in a number of different functional categories with the most occurring in the Biological Process GO domain

Gene Ontology (GO) annotations of differentially expressed genes were collected from the WormBase ParaSite Biomart [20]. In order to better understand the functional distribution of the genes at a global level, WEGO software was used to classify the terms into high level categories. The 1641 DEGs were annotated to 40 functional groups (Fig. 2). 10 groups were contained within the Cellular Component root category, 10 in the Molecular Function root category, and 20 in the Biological Process root. Within Cellular Component, the groups containing the most genes were cell (GO:0005623), organelle (GO:0043226) and macromolecular complex (GO:0032991). Within Molecular Function, a large proportion of genes were categorized to catalytic activity (GO:0003824), binding (GO:0005488), structural molecule activity (GO:0005198) and transporter activity (GO:0005215). The categories of metabolic process (GO:0008152), cellular process (GO:0009987), reproduction (GO:0000003), developmental process (GO:0032502) and growth (GO:0040007) contained a large number of genes within the root level Biological Process category.

To further explore which, if any, GO terms were overrepresented, gene set enrichment analysis was performed using the topGO R package [21]. Categories of significance were found for each of the root GO categories (Table 1). A total of 14 GO groups were found to be enriched at an unadjusted *p*-value < 0.05. The groups include metabolic process, cell cycle, body morphogenesis, carbohydrate metabolic process, cytoplasm, endoplasmic reticulum, translation elongation factor activity and acid phosphatase activity. The most significantly enriched groups (unadjusted *p* < 0.001) were proteolysis (GO:0006508) and structural constituents of the ribosome (GO:0003735).

**Table 1:** Significantly enriched GO terms in DEGs

GO Domain	Go Accession	GO Term	Number of Genes (Significant/Total Annotated)	*p*-value (Fisher’s Exact Test)
Molecular Function	GO:0003735	structural constituent of ribosome	39/52	<1E-20
Biological Process	GO:0006508	proteolysis	26/103	1.07E-05
Molecular Function	GO:0003746	translation elongation factor activity	3/3	0.00167
Molecular Function	GO:0003993	acid phosphatase activity	5/10	0.00351
Biological Process	GO:0010171	body morphogenesis	3/4	0.00744
Biological Process	GO:0008152	metabolic process	105/673	0.01048
Molecular Function	GO:0016788	hydrolase activity, acting on ester bonds	10/35	0.02239
Molecular Function	GO:0004129	cytochrome-c oxidase activity	3/6	0.02535
Molecular Function	GO:0016810	hydrolase activity, acting on carbon-nitrogen (but not peptide) bonds	4/8	0.03872
Molecular Function	GO:0004359	glutaminase activity	2/3	0.039
Molecular Function	GO:0008137	NADH dehydrogenase (ubiquinone) activity	2/3	0.039
Cellular Component	GO:0005737	cytoplasm	19/97	0.0408
Biological Process	GO:0005975	carbohydrate metabolic process	14/66	0.04955
Cellular Component	GO:0005789	endoplasmic reticulum	2/3	0.0496

### KEGG annotations

**Fig. 3:**
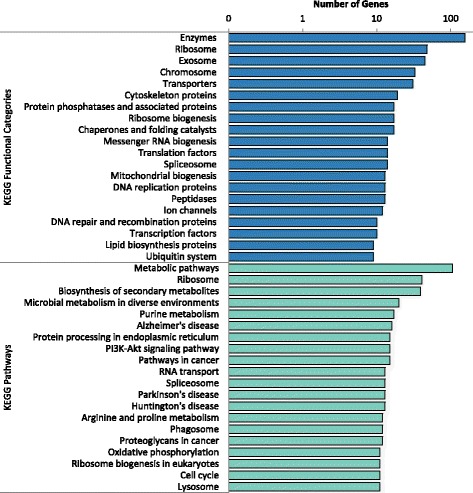
KEGG functional and pathway annotations for DEGs. Using the peptide sequences of DEGs, functional and pathway annotations were found using the KEGG Automatic Annotation Server and eggNOG, respectively. Annotations covered a variety of different pathway and functional annotations. The 20 most represented categories by KAAS and eggNOG annotation are shown

In addition to annotating DEGs with GO terms, Kyoto Encyclopedia of Genes and Genomes KEGG functional categories and pathways were also found. KEGG functional categories covered enzymes (Enzymes, Protein phosphatases and associated proteins, Peptidases, Chaperones and folding catalysts) and nucleic acid machinery (Ribosome, Chromosome, Ribosome biogenesis, Messenger RNA biogenesis and DNA replication proteins). Similarly, KEGG pathway annotation identified pathways involved in ribosome biogenesis and protein processing pathways (Fig. 3).

### Comparison of DEGs with different nematode clades and representative species

**Fig. 4:**
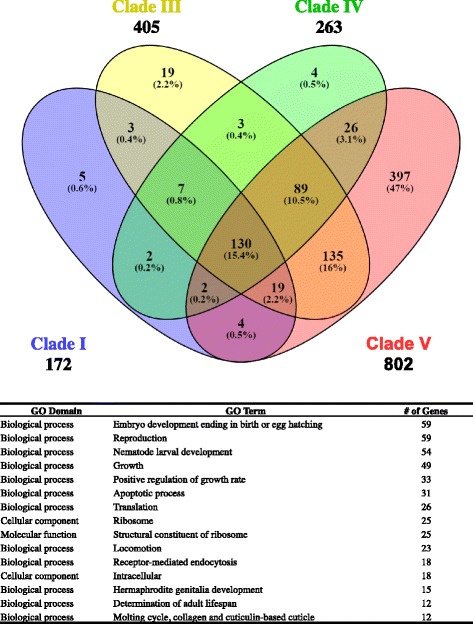
Distribution of DEGs across nematode clades. Overlaps between alignments between *H. bacteriophora* and different nematode clades were found using Venny 2.1. The majority of alignments were exclusive to Clade V (excluding *H. bacteriophora*). However a number of genes were shared between all Clades and single genes were annotated to multiple GO categories. The top fifteen GO categories for the 130 genes shared by all clades are shown in the table below the diagram

In order to identify a set of common PN genes expressed during the early stages of infection, a series of data filters were used on genes identified as significantly changed (FC ≥ 2 or FC ≤ −2, *p* < 0.05) during differential expression analysis. Protein sequences of DEGs were blasted against all sequenced nematode genomes in each clade (*H. bacteriophora* was excluded from the Clade V blast). Alignments were considered matches if the percent identity was greater than or equal to 60 and the *e*-value was less than 0.00005. The greatest number of shared genes (802) was between *H. bacteriophora* and Clade V. A total of 130 genes were found to be common between *H. bacteriophora* and the nematodes of Clades I, III, IV and V (Fig. 4). GO annotation was performed on the genes within the common pool. The genes were primarily categorized to groups involving reproduction, development and growth.

**Fig. 5:**
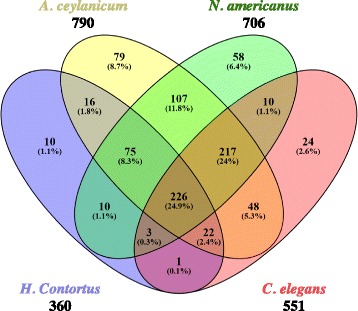
Distribution of DEGs across clade V parasitic nematodes and *C. elegans*. Overlaps between alignments between *H. bacteriophora* and different Clade V nematodes were found using Venny 2.1. While 226 genes were found to be common between *C. elegans* and the parasitic nematodes surveyed, a subset of 75 genes was shared among parasitic nematodes

Due to the DEGs predominantly aligning with Clade V nematodes, further comparisons between parasites and *H. bacteriophora* were made using only Clade V nematodes. A second blast of the 1641 genes was performed against the genomes of *C. elegans*, *Ancylostoma ceylanicum*, *Necator americanus* and *Haemonchus contortus*. The same criteria used for the blast against nematode clades were used to determine if alignments were matches with the above nematodes. After analysis, 551 genes were hits for *C. elegans* genes, 790 genes matched *A. ceylanicum*, 706 genes matched *N. americanus*, and 363 genes matched *H. contortus*. 226 of the genes were held in common by the four nematode species and *H. bacteriophora*, while 75 genes were shared between the parasitic nematodes *A. ceylanicum*, *H. contortus* and *N. americanus* (Fig. 5). Of the 75 genes, only 45 had annotation data available in the ParaSite Biomart database. The GO terms, InterPro ID and other protein information for the 45 matches are shown in Table 2.

**Table 2:** Common genes of clade V parasitic nematodes

Gene stable ID	GO term accession	GO term name	InterPro ID	InterPro short description	Signal Present	Coiled coils	Transmembrane domain
Hba_00415	GO:0016021	integral component of membrane	IPR010761	Clc_prot-like	-	-	TMhelix
Hba_03156	GO:0050660	flavin adenine dinucleotide binding	IPR004113	FAD-linked_oxidase_C	-	-	TMhelix
Hba_05467	GO:0006810	transport	IPR003439	ABC_transporter-like	-	-	TMhelix
Hba_06397	GO:0005975	carbohydrate metabolic process	IPR001223	Glyco_hydro18cat	-	-	-
Hba_06467	GO:0007017	microtubule-based process	IPR001372	Dynein_light_chain_typ-1/2	-	-	-
Hba_06770	GO:0005975	carbohydrate metabolic process	IPR001360	Glyco_hydro_1	-	-	-
Hba_06875	GO:0005921	gap junction	IPR000990	Innexin	-	-	TMhelix
Hba_06876	GO:0005921	gap junction	IPR000990	Innexin	-	-	TMhelix
Hba_06877	GO:0005921	gap junction	IPR000990	Innexin	-	-	-
Hba_07556	GO:0006810	transport	IPR002259	Eqnu_transpt	-	-	TMhelix
Hba_07557	GO:0006810	transport	IPR002259	Eqnu_transpt	-	-	TMhelix
Hba_08573	GO:0016020	membrane	IPR006029	Neurotrans-gated_channel_TM	-	-	TMhelix
Hba_08939	GO:0004871	signal transducer activity	IPR015898	G-protein_gamma-like_dom	-	-	-
Hba_09140	GO:0016020	membrane	IPR007265	COG_su3	-	-	TMhelix
Hba_09704	GO:0005515	protein binding	IPR001611	Leu-rich_rpt	-	Coil	TMhelix
Hba_09919	GO:0006334	nucleosome assembly	IPR002164	NAP_family	-	-	-
Hba_10371	GO:0005524	ATP binding	IPR004523	Asp-tRNA_synthase	-	-	-
Hba_10698	GO:0040010	positive regulation of growth rate	IPR026847	VPS13	-	-	TMhelix
Hba_11850	GO:0003677	DNA binding	IPR000536	Nucl_hrmn_rcpt_lig-bd_core	-	-	-
Hba_12267	GO:0000138	Golgi trans cisterna	IPR007258	Vps52	-	-	TMhelix
Hba_12451	GO:0005515	protein binding	IPR000626	Ubiquitin_dom	-	Coil	-
Hba_13253	GO:0005515	protein binding	IPR000210	BTB/POZ-like	-	-	-
Hba_13349	GO:0016021	integral component of membrane	IPR011701	MFS	-	-	TMhelix
Hba_13917	GO:0016020	membrane	IPR005027	Glyco_trans_43	-	-	-
Hba_14055	GO:0016020	membrane	IPR002159	CD36	-	-	TMhelix
Hba_14609	GO:0009792	embryo development ending in birth or egg hatching	-	-	-	-	-
Hba_14679	GO:0005515	protein binding	IPR001611	Leu-rich_rpt	SignalP-noTM	-	-
Hba_14742	GO:0001104	RNA polymerase II transcription cofactor activity	IPR019145	Mediator_Med10	-	Coil	-
Hba_14927	GO:0006189	‘de novo’ IMP biosynthetic process	IPR000031	PurE_dom	-	-	-
Hba_15073	GO:0006355	regulation of transcription, DNA-templated	IPR000536	Nucl_hrmn_rcpt_lig-bd_core	-	-	-
Hba_15994	GO:0005634	nucleus	IPR002999	Tudor	-	-	-
Hba_17350	GO:0006270	DNA replication initiation	IPR003874	CDC45	-	-	-
Hba_17394	GO:0006508	proteolysis	IPR001969	Aspartic_peptidase_AS	SignalP-noTM	-	-
Hba_17412	GO:0016020	membrane	IPR000731	SSD	-	-	TMhelix
Hba_18346	GO:0006457	protein folding	IPR002777	PFD_beta-like	-	Coil	-
Hba_18755	GO:0008146	sulfotransferase activity	IPR000863	Sulfotransferase_dom	-	-	-
Hba_19983	GO:0003777	microtubule motor activity	IPR013594	Dynein_heavy_dom-1	-	Coil	-
Hba_20009	GO:0009792	embryo development ending in birth or egg hatching	IPR007051	CHORD	-	-	-
Hba_20121	GO:0009792	embryo development ending in birth or egg hatching	-	-	-	-	-
Hba_20127	GO:0055114	oxidation-reduction process	IPR008972	Cupredoxin	-	-	-
Hba_20282	GO:0016021	integral component of membrane	IPR008855	TRAP-delta	SignalP-noTM	-	-
Hba_20782	GO:0005515	protein binding	IPR003172	ML_dom	-	-	-
Hba_20870	GO:0016020	membrane	IPR003492	Battenin_disease_Cln3	-	-	TMhelix
Hba_21214	GO:0004568	chitinase activity	IPR000726	Glyco_hydro_19_cat	SignalP-noTM	-	-
Hba_21297	GO:0003723	RNA binding	IPR001040	TIF_eIF_4E	-	-	-

### Identification of genes activated during the initial stages of host infection

Upregulated DEGs were further analyzed to determine if any genes could be identified as highly conserved PN genes. Out of the 239 differentially expressed up regulated genes (*p* < 0.05, FDR < 0.05, FC ≥ 2), 52 genes (21.8%) were found to contain a classic or non-classical signal peptide, and to be non-orthologous to *C. elegans* but orthologous to *A. ceylanicum* (Bioproject PRJNA231479). Of these 52 *H. bacteriophora* genes, 17 were determined to be initial activation candidates due to either the identification of proteins and/or molecular mechanisms believed to be involved in parasitism (by GO grouping and InterPro ID) or the lack of any identifiable protein motifs (Table 3). Of the 17 genes, 16 were identified as having enzymatic motifs, including both hydrolases and kinases. The remaining gene, Hba_13349, was identified as being involved in membrane transport.

**Table 3:** Highly conserved clade V parasitic nematode genes upregulated in *H. bacteriophora* incubated in hemolymph plasma

Gene ID	GO Accession	GO Name	InterPro ID	InterPro Description
Hba_05422	GO:0005515	Protein binding	IPR011105	Cell_wall_hydrolase_SleB
Hba_06426	GO:0004190	Aspartic-type endopeptidase activity	IPR021109	Peptidase_aspartic_dom
Hba_07292	GO:0016758	Transferase activity, transferring hexosyl groups	IPR002213	UDP_glucos_trans
Hba_07973	GO:0004181	Metallocarboxypeptidase activity	IPR000834	Peptidase_M14
Hba_08473	GO:0008483	Transaminase activity	IPR005814	Aminotrans_3
Hba_11636	GO:0004185	Serine-type carboxypeptidase activity	IPR001563	Peptidase_S10
Hba_11637	GO:0004185	Serine-type carboxypeptidase activity	IPR001563	Peptidase_S10
Hba_13000	GO:0008236	Serine-type peptidase activity	IPR008758	Peptidase_S28
Hba_13072	GO:0008378	Galactosyltransferase activity	IPR002659	Glyco_trans_31
Hba_13349	GO:0055085	Transmembrane transport	IPR020846	MFS_dom
Hba_13477	GO:0004672	Protein kinase activity	IPR011009	Kinase-like_dom
Hba_14122	GO:0003993	Acid phosphatase activity	IPR029033	His_PPase_superfam
Hba_15308	GO:0008234	Cysteine-type peptidase activity	IPR013128	Peptidase_C1A
Hba_17215	GO:0003824	Catalytic activity	IPR031319	A-amylase_C
Hba_19909	GO:0003796	Lysozyme activity	IPR008597	Destabilase
Hba_20878	GO:0055085	Transmembrane transport	IPR020846	MFS_dom
Hba_20939	GO:0003993	Acid phosphatase activity	IPR029033	His_PPase_superfam

## Discussion

Parasitic nematode infections continue to pose a considerable burden to human health. However, the obligate need for a vertebrate host has made efforts to study the molecular mechanisms of parasitic infection intractable and highlights the need for an analogous model nematode. Due to its life cycle and recent studies, the possibility of developing and using *H. bacteriophora* as a model for parasitic infections has grown. Basic advances in the development of a molecular toolbox and the publication of its genome provides a way for manipulating and identifying the function of parasitic genes [5, 8, 13, 19]. In order to create a smaller list of genes involved in infection for future investigation, we performed RNA-seq on *H. bacteriophora* strain TT01 IJs incubated in hemolymph plasma for 9 h. After differential expression analysis, a total of 1641 genes were identified as being differentially expressed after a 9 h exposure to hemolymph plasma. Hemolymph plasma from *Manduca sexta* was selected as an activation media because *M. sexta* is a natural host, and its large size facilitates extraction of large quantities of hemolymph. Previous studies identified hemolymph soaking as a valid method for inducing synchronous activation of entomopathogenic IJs, while also allowing for the mass activation of IJs necessary to produce enough high quality RNA for RNA-seq [19, 22–24]. Additionally, our pilot studies used to determine the maximum concentration of IJs per milliliter hemolymph plasma showed IJs develop into J4s within 48 h of soaking and continue to adults by 72 h (data not shown). After 1 week of soaking in hemolymph plasma, multiple generations of nematodes were present.

Assignment of GO terms to DEGs categorized 769 of the genes to probable functions. Some of the most represented functional categories included reproduction, reproductive process, developmental process, binding and catalytic activity. By checking for overrepresented GO terms, we found a number of genes involved in proteolysis, acid phosphatase activity, NADH dehydrogenase (ubiquinone) activity and hydrolases, as well as sequences associated with ribosomal constituents and translation elongation factors. Also, a number of overrepresented terms were also found in categories related to development such as body morphogenesis, carbohydrate metabolism and metabolic processes, consistent with the resumption of development by the IJs.

KEGG functional categories and pathways were also assigned to DEGs. KEGG assignments showed similar categories as the overrepresented GO terms. A large number of sequences were categorically assigned ribosome, ribosome biogenesis, messenger RNA biogenesis, cytoskeleton proteins and chaperones and folding catalysts suggesting upregulation in protein production is necessary to exit the developmentally arrested dauer stage. Also seen in the KEGG functional categories again are protein phosphatases and associated proteins, ubiquitin system and peptidases. While the KEGG and GO analysis does not provide enough resolution to identify parasitic genes directly, the recurrence and overrepresentation of proteolytic enzymes suggests some of these genes may be involved in the production of proteins/catalysts necessary for parasitism. This idea is further supported by an increase in the transporters category since gene products important for PN-host interactions are likely secreted into host tissues. The KEGG pathway assignments mirror the KEGG functional categories by also showing a number of genes involved in biosynthesis of secondary metabolites, protein processing in endoplasmic reticulum, ribosome biogenesis and lysosome.

In an attempt to identify common parasitic genes, a series of protein blasts were performed against each nematode clade and also against representative nematodes of Clade V. Blasting the protein sequences of the *H. bacteriophora* DEGs against all nematodes included in Clades I, III, IV and V (not including *H. bacteriophora*) returned 172, 405, 263 and 802 gene hits respectively. As expected, the majority of hits (397 genes) were found only in Clade V due to *H. bacterophora’s* close phylogenetic relationship with its members. Interestingly, while there is overlap with Clades III and IV, there are 405 hits with Clade III and only 263 with Clade IV. Given their phylogenetic relationship to Clade V, hits would be expected to decrease as the distance between clades increases. The blast searches by clade also suggest a subset of “pan-nematode” genes (130) which are shared by all four of the clades surveyed. GO term assignment identifies these genes as being involved in embryo development, reproduction, nematode larval development, life cycle development and growth. Given the apparent conservation of genes related to reproduction and development, it is possible these genes represent the basic genetic needs for the reproduction and development of nematodes.

Given the relatively large number of hits with Clade V nematodes, another protein blast was performed in order to further investigate the presence of a set of PN genes. For this reason, this Clade V blast was limited to *A. ceylanicum*, *H. contortus*, *N. americanus* and *C. elegans*. While *C. elegans* is not a parasitic nematode, given the deep understanding and robust annotation of its genome, it was included in order to further separate Clade V nematode genes from the highly conserved genes of Clade V PNs. While a number of DEGs were found to hit each nematode surveyed, 75 genes were found to be shared by *A. ceylanicum*, *H. contortus* and *N. americanus*. Of these 75 genes, 30 have no GO, InterPro ID or protein motifs assigned in the ParaSite Biomart database. The 45 remaining genes contain a variety of different functional categories, but are predominantly represented by categories involved in growth and development. However, some proteins of possible parasitic interest are present. Most notably, 4 genes (Hba_14679, Hba_17394, Hba_20282 and Hba_21214) contain signal peptides with no transmembrane region. Hba_14679 contains a protein motif for leucine-rich repeats, which serve as structural supports for protein-protein interactions [25]. While a wide variety of proteins make use of these supports, given the presence of a signaling peptide, the product of Hba_14679 may play a role in host-parasite interactions external to the worm. Hba_20282 contains a motif for the delta subunit precursor of a translocon. While the exact function of this protein is unknown, translocons are known to transport peptides across membranes. Within eukaryotes translocons are commonly used to transfer polypeptides into the endoplasmic reticulum [26]. However, in prokaryotes, translocons can be assembled to export virulence factors outside of the cell [27, 28] While this protein may serve no other purpose than shuttling molecules into the endoplasmic reticulum, its conservation between 4 different parasites suggests that it may be needed for the transport of parasitism factors.

Hba_17394 and Hba_21214 are both enzymes which may be externally secreted since they both contain signal peptides. Hba_17394 is identified to have an aspartic peptidase motif. While further functional investigation is necessary for an exact identity, aspartic peptidases are known to have a range of functions from the digestion of peptides to the production of active proteins from precursor proteins [29, 30]. Within parasites, such a peptidase could be secreted for use as a virulence factor. Hba_21214 contains a protein motif for a glycoside hydrolase, family 19. Glycoside hydrolases are chitinases which break down glycosidic bonds between carbohydrates [31]. Chitinases are most commonly used as defense mechanisms to break down the cell walls of fungal and insect pathogens [32–34]. Within *H. bacteriophora*, the presence of a chitinase seems obligate since the digestion of chitin would be necessary upon infection of a host insect. However, given its presence across several parasitic nematodes, it is possible a secreted chitinase may serve a more general role in parasitism [35].

Given that any likely parasitism genes would potentially be upregulated upon exposure to a host, further analyses was carried out to identify any upregulated DEGs conserved within parasitic nematodes by comparison to *A. ceylanicum* (Bioproject PRJNA231479). Out of 861 upregulated DEGs, 239 had a false discovery rate less than 0.05. Of these 239 genes, 17 genes were identified as being potential conserved parasitism genes due to being orthologous to *A. ceylanicum* and containing a signal peptide without a transmembrane region and lacking orthology with free-living nematodes. The majority of these genes (16) were identified to have enzymatic protein motifs by InterPro ID. These enzymes include peptidases (7), phosphatases (2) and a kinase. The non-catalytic peptide is identified as having a transmembrane transport motif. While individually, the peptidases listed in Table 2 could be present for a number of different biological processes, their inclusion as secreted peptides orthologous to *A. ceylanicum* and non-orthologous with *C. elegans* suggests they are conserved parasitism genes. Furthermore, it is possible these genes are involved in the mechanisms of parasitism either as virulence factors or modulators of the host immune system.

## Conclusions

Taken together, our RNA-Seq analysis reveals transcriptional changes in the regulation of a large number of genes, most of which have not been shown previously to play a role in parasitic responses. A significant proportion of these genes are conserved amongst closely related parasitic nematodes, suggesting the identification of a group of parasitism factors within nematodes. These genes provide ideal candidates for functional characterization using recently developed tools in *H. bacteriophora* to dissect the contribution of these genes in infection. While our selection criteria potentially misses some genes of interest, future studies using these candidates, in addition to further data-mining of our sequencing data, will not only provide functional insight into the process of nematode parasitism but may also shed light on the evolution of parasitism within nematodes as our data also reveals the existence of conserved genes amongst PN that are upregulated during early infection.

## Methods

### Culturing of *Heterorhabditis bacteriophora* TT01

*Heterorhabditis bacteriophora* strain TT01 was maintained by infecting *Galleria mellonella* larvae. Briefly, 20 *G. mellonella*, approximately 5^th^ to 6^th^ instar, were placed onto 8.5 cm filter papers held in 9 cm petri dishes. Approximately 100 IJs per larva were added to the filter paper (~1 mL total volume) and placed in a resealable bag in the dark at room temperature for incubation. After 10 days, infected insects turned brick red in color and were transferred to a White trap [36] containing distilled water with 0.01% Tween 20. Thirteen to fifteen days post-infection the IJs began to emerge from the carcasses and entered the liquid. After 7 days on the trap, IJs were collected and transferred to sterile culture flasks until use. IJs were used within 3 weeks of collection.

### Hemolymph plasma extraction from *Manduca sexta*

Five 5^th^ instar *Manduca sexta* larvae were placed on ice for 15 min. The posterior end of the insect was sterilized with an alcohol wipe and using aseptic technique, an incision was made at the distal end of the insect’s horn. The insect was gently exsanguinated by squeezing, and the hemolymph was collected in a sterile 2 mL microfuge tube on ice. To inhibit melanization, a solution of 20 mM phenylthiocarbamide in phosphate buffered saline was immediately added to the hemolymph at a final concentration of 0.33 mM. In order to further inhibit melanization of the hemolymph during incubation, the hemolymph was centrifuged at 4 °C for 5 min at 4000 × g to precipitate the hemocytes. The resulting hemolymph plasma was diluted 1:1 with Ringer’s solution (100 mM NaCl, 1.8 mM KCl, 2 mM CaCl_2_, 1 mM MgCl_2_, 5 mM HEPES, pH 6.9) and filtered through a 0.45 μm syringe filter. Hemolymph plasma samples were stored at −80 °C until needed.

### Infective juvenile incubation in hemolymph plasma solution

Approximately 50,000 *H. bacteriophora* TT01 infective juveniles were surface-sterilized with 10 mL of 3% commercial bleach in Ringer’s solution (hypochlorite final concentration of 0.26%) for 5 min. The nematodes were centrifuged at 500 × g for 2 min at room temperature. The resulting pellet was washed three times with 10 mL sterile Ringer’s solution and centrifuged. The nematode pellet was either suspended in 2 mL of hemolymph plasma solution and transferred to a 12.5 cm^2^ tissue flask or reserved as a 0 h control. Tissue flasks were incubated for 9 h in the dark at 27 °C with shaking at 300 RPM. The flask contents were transferred to a sterile 15 mL conical tube and centrifuged at 1100 × g at room temperature for 2 min. The pelleted nematodes were washed a total of three times with 10 mL sterile Ringer’s solution with centrifugation at 1100 × g between washes. The pellet was suspended in 500 μL ice-cold Trizol reagent (Thermo Fisher) and transferred to a 1.5 mL microfuge tube containing 1.4 g of 0.5 mm zirconium oxide beads. Tubes were placed in a Bullet Blender Blue (NextAdvance, Averill Park, NY) and lysed (3 cycles of 5 min at speed 10, with a 2 min rest on ice between each cycle). The resulting homogenate was transferred to a clean microfuge tube and an additional 500 μL of Trizol was added. RNA purification was carried out using the Trizol RNA Plus Purification Kit according to the manufacturer’s protocol and the PureLink DNase protocol was used to remove contaminating DNA. RNA from 0 h IJs was isolated as above, with the exception that RNA extraction began immediately after washing the surface sterilized IJs. RNA samples were analyzed for quantity and quality using an Agilent 2100 Bioanalyzer with the RNA 6000 Nano Kit. Samples with a RIN greater than 9.0 were sent to the Institute for Genome Sciences (University of Maryland School of Medicine) for RNA-Seq using the Illumina HiSeq4000 platform with 150 bp, paired-end sequencing. RNA-seq libraries were prepared using the TruSeq RNA Sample Prep Kit (Illumina, San Diego, CA). Double-stranded cDNA was ligated to seven indexed nucleotide adapters and purified between enzymatic reactions. Library size selection was performed using SPRIselect beads (Beckman Coulter Genomics, Danvers, MA).

### RNA-Seq analysis using subread and edgeR

Illumina adapter sequences, leading and trailing bases and low quality base reads (Phred-64 score < 15) were removed from RNA-Seq data using Trimmomatic (version 0.33) [37]. After trimming, only reads greater than or equal to 25 bases were retained. Reads were then mapped to the *H. bacteriophora* genome (Bioproject PRJNA13977) [5] using subread-aligner (version 1.5.0-p1) and the *H. bacteriophora* genome [38]. The aligned reads were counted using featureCounts and differential expression analysis was performed using the edgeR package’s quasi-likelihood F test [39, 40]. Genes were considered differentially expressed if they had a *p*-value < 0.05 and a fold change ≥ 2 or a fold change ≤ −2.

### qRT-PCR validation of RNA-seq

Selected DEGs were validated by qRT-PCR using the Brilliant II SYBR Green QRT-PCR 1-step Master Mix (Agilent Technologies) and CFX96 Real-Time System (Bio-Rad Laboratories) with *Hba-rpl-32* as an expression control. Primer efficiencies for primers targeting the selected DEGs were determined using serial dilutions (0.001 to 100 ng total RNA) of RNA extracted from untreated IJs, while the *rpl-32* primers used were previously published [8]. PCR reactions were carried out using the manufacturer’s suggested two-step protocol with an annealing temperature of 55 °C and a dissociation curve performed at the end of the run. In addition to validating expression in samples used for RNA-seq, changes in expression were also verified in an additional set of samples prepared by incubating IJs in hemolymph plasma, as described above. Differential expression was measured using the ΔΔC_T_ method, using matched, untreated IJs as relative controls.

### Annotation and analysis of differentially expressed genes

DEGs were annotated and further analyzed using a variety of bioinformatic tools and databases. Peptide sequences, *C. elegans* orthologues, GO terms, InterProScan IDs, transmembrane regions, coiled coils and classical secretory sequences were gathered from the WormBase ParaSite Biomart database for *H. bacteriophora* [41]. Non-classical secretory peptides were found using SecretomeP v1.0f [42]. In order to better understand global gene ontology classifications, terms were condensed into higher functional categories using WEGO [43]. To determine if any GO terms were significantly over-represented, an enrichment analysis was performed with the topGO R package using Fisher’s exact test and the default hybrid classic/elim algorithm [21]. Annotation of KEGG pathways was performed using the peptide sequences of the DEGs and eggNOG verion 4.5 [44]. KEGG functional annotations were also gathered using the same peptide sequences and the KEGG Automatic Annotation Server [45]. Orthology between DEGs and other nematodes was examined using blastp and the genomes available for Clade I, Clade III, Clade IV and Clade V (excluding *H. bacteriophora*) nematodes. Blast results were considered hits if the aligned sequence had percent identity greater than 60% and an *e*-value less than 0.00005. Overlaps between alignments from different search targets (e.g. Clades I, III, IV and V) were found and graphed using Venny 2.1 [46].

## Additional file

Additional file 1: Table S1. Summary of Illumina sequencing reads. **Figure S1** RNA-seq Analysis Pipeline. Pipeline used to collect, trim and analyze RNA-seq data. RNA sequencing reactions were performed by the Institute of Genome Sciences (University of Maryland School of Medicine) using high quality total RNA obtained from IJs incubated in hemolymph plasma (9 h) or Ringer’s solution (0 h). The resulting reads were trimmed, screened for quality and mapped to the *H. bacteriophora* reference genome. Expression analysis for DEGs was performed using the edgeR package (PDF 634 kb)

## Abbreviations

DEG: Differentially expressed gene
EPN: Entomopathogenic nematode
GO: Gene Ontogeny
IJ: Infective juvenile
KEGG: Kyoto Encyclopedia of Genes and Genomes
PN: Parasitic nematode
qRT-PCR: Quantitative reverse transcriptase-polymerase chain reaction

## References

[1] Hotez PJ, Fenwick A, Savioli L, Molyneux DH (2009). Rescuing the bottom billion through control of neglected tropical diseases. Lancet.

[2] John M (2014). Hawdon: Controlling Soil-Transmitted Helminths: Time to Think Inside the Box?. J Parasitol.

[3] Erb KJ (2009). Can helminths or helminth-derived products be used in humans to prevent or treat allergic diseases?. Trends Immunol.

[4] Castillo JC, Reynolds SE, Eleftherianos I (2011). Insect immune responses to nematode parasites. Trends Parasitol.

[5] Bai X, Adams BJ, Ciche TA, Clifton S, Gaugler R, Kim K, Spieth J, Sternberg PW, Wilson RK, Grewal PS (2013). A lover and a fighter: the genome sequence of an entomopathogenic nematode Heterorhabditis bacteriophora. PLoS One.

[6] Ciche T. The biology and genome of Heterorhabditis bacteriophora (February 20, 2007), WormBook, ed. The C. elegans Research Community, WormBook. doi:10.1895/wormbook.1.135.1. 10.1895/wormbook.1.135.1 PMC4781484 18050499

[7] Hallem EA, Rengarajan M, Ciche T, Sternberg PW (2007). Nematodes, Bacteria, and Flies: A Tripartite Model for Nematode Parasitism. Curr Biol.

[8] Ratnappan R, Vadnal J, Keaney M, Eleftherianos I, O’Halloran D, Hawdon JM (2016). RNAi-mediated gene knockdown by microinjection in the model entomopathogenic nematode Heterorhabditis bacteriophora. Parasit Vectors.

[9] Ciche TA, Darby C, Ehlers R, Forst S, Goodrich-Blair H (2006). Dangerous liaisons: The symbiosis of entomopathogenic nematodes and bacteria. Biol Control.

[10] Hotez P, Hawdon J, Schad GA (1993). Hookworm larval infectivity, arrest and amphiparatenesis: the Caenorhabditis elegans daf-c paradigm. Parasitol Today.

[11] Crook M (2014). The dauer hypothesis and the evolution of parasitism: 20 years on and still going strong. Int J Parasitol.

[12] Hashmi S, Abu Hatab MA, Gaugler RR (1997). GFP : green fluorescent protein a versatile gene marker for entomopathogenic nematodes. Fundam Appl Nematol.

[13] Ciche TA, Sternberg PW (2007). Postembryonic RNAi in Heterorhabditis bacteriophora: a nematode insect parasite and host for insect pathogenic symbionts. BMC Dev Biol.

[14] Hashmi S, Hashmi G, Gaugler R (1995). Genetic Transformation of an Entomopathogenic Nematode by Microinjection. J Invertebr Pathol.

[15] Castillo JC, Shokal U, Eleftherianos I (2012). A novel method for infecting Drosophila adult flies with insect pathogenic nematodes. Virulence.

[16] Eleftherianos I, Ffrench-Constant RH, Clarke DJ, Dowling AJ, Reynolds SE (2010). Dissecting the immune response to the entomopathogen Photorhabdus. Trends Microbiol.

[17] Eleftherianos I, Joyce S, Ffrench-Constant RH, Clarke DJ, Reynolds SE (2010). Probing the tri-trophic interaction between insects, nematodes and Photorhabdus. Parasitology.

[18] Bai X, Adams BJ, Ciche TA, Clifton S, Gaugler R, Hogenhout SA, Spieth J, Sternberg PW, Wilson RK, Grewal PS (2009). Transcriptomic analysis of the entomopathogenic nematode Heterorhabditis bacteriophora TTO1. BMC Genomics.

[19] Moshayov A, Koltai H, Glazer I (2013). Molecular characterisation of the recovery process in the entomopathogenic nematode Heterorhabditis bacteriophora. Int J Parasitol.

[20] Howe KL, Bolt BJ, Cain S, Chan J, Chen WJ, Davis P, Done J, Down T, Gao S, Grove C, Harris TW, Kishore R, Lee R, Lomax J, Li Y, Muller H, Nakamura C, Nuin P, Paulini M, Raciti D, Schindelman G, Stanley E, Tuli MA, Van Auken K, Wang D, Wang X, Williams G, Wright A, Yook K, Berriman M, Kersey P, Schedl T, Stein L, Sternberg PW (2016). WormBase 2016: expanding to enable helminth genomic research. Nucleic Acids Res.

[21] Alexa A, Rahnenfuhrer J (2016). topGO: Enrichment Analysis for Gene Ontology.

[22] Hao Y, Montiel R, Nascimento G, Toubarro D, Simoes N (2008). Identification, characterization of functional candidate genes for host–parasite interactions in entomopathogenetic nematode Steinernema carpocapsae by suppressive subtractive hybridization. Parasitol Res.

[23] Ciche TA, Ensign JC (2003). For the Insect Pathogen Photorhabdus luminescens, Which End of a Nematode Is Out?. Appl Environ Microbiol.

[24] Hao Y, Montiel R, Abubucker S, Mitreva M, Simões N (2010). Transcripts analysis of the entomopathogenic nematode Steinernema carpocapsae induced in vitro with insect haemolymph. Mol Biochem Parasitol.

[25] Kedzierski L, Montgomery J, Bullen D, Curtis J, Gardiner E, Jimenez-Ruiz A, Handman E (2004). A leucine-rich repeat motif of Leishmania parasite surface antigen 2 binds to macrophages through the complement receptor 3. J Immunol.

[26] Holthuis JC, van Riel MC, Martens GJ (1995). Translocon-associated protein TRAP delta and a novel TRAP-like protein are coordinately expressed with pro-opiomelanocortin in Xenopus intermediate pituitary. Biochem J.

[27] Atkinson S, Williams P (2016). Yersinia virulence factors - a sophisticated arsenal for combating host defences. F1000Res.

[28] Coombes BK, Finlay BB (2005). Insertion of the bacterial type III translocon: not your average needle stick. Trends Microbiol.

[29] Rawlings ND, Barrett AJ (1993). Evolutionary families of peptidases. Biochem J.

[30] Sojka D, Hartmann D, Bartosova-Sojkova P, Dvorak J (2016). Parasite Cathepsin D-Like Peptidases and Their Relevance as Therapeutic Targets. Trends Parasitol.

[31] Davies G, Henrissat B (1995). Structures and mechanisms of glycosyl hydrolases. Structure.

[32] Hamid R, Khan MA, Ahmad M, Ahmad MM, Abdin MZ, Musarrat J, Javed S (2013). Chitinases: An update. J Pharm Bioallied Sci.

[33] Punja ZK, Zhang YY (1993). Plant chitinases and their roles in resistance to fungal diseases. J Nematol.

[34] Bordon Y (2014). Parasite immunity: chitinase-like proteins smoke out worms. Nat Rev Immunol.

[35] Tachu B, Pillai S, Lucius R, Pogonka T (2008). Essential Role of Chitinase in the Development of the Filarial Nematode Acanthocheilonema viteae. Infect Immun.

[36] Lindegren JE, Valero KA, Mackey BE (1993). Simple In Vivo Production and Storage Methods for Steinernema carpocapsae Infective Juveniles. J Nematol.

[37] Bolger AM, Lohse M, Usadel B (2014). Trimmomatic: a flexible trimmer for Illumina sequence data. Bioinformatics.

[38] Liao Y, Smyth GK, Shi W (2013). The Subread aligner: fast, accurate and scalable read mapping by seed-and-vote. Nucleic Acids Res.

[39] Liao Y, Smyth GK, Shi W (2014). featureCounts: an efficient general purpose program for assigning sequence reads to genomic features. Bioinformatics.

[40] Robinson MD, McCarthy DJ, Smyth GK (2010). edgeR: a Bioconductor package for differential expression analysis of digital gene expression data. Bioinformatics.

[41] Howe KL, Bolt BJ, Shafie M, Kersey P, Berriman M. WormBase ParaSite - a comprehensive resource for helminth genomics. Mol Biochem Parasitol. 2016;(16)30160–8. 10.1016/j.molbiopara.2016.11.005 PMC5486357 27899279

[42] Bendtsen JD, Jensen LJ, Blom N, Von Heijne G, Brunak S (2004). Feature-based prediction of non-classical and leaderless protein secretion. Protein Eng Des Sel.

[43] Ye J, Lin F, Zheng H, Zhang Y, Chen J, Zhang Z, Wang J, Li S, Li R, Bolund L, Wang J (2006). WEGO: a web tool for plotting GO annotations. Nucleic Acids Res.

[44] Huerta-Cepas J, Szklarczyk D, Forslund K, Cook H, Heller D, Walter MC, Rattei T, Mende DR, Sunagawa S, Kuhn M, Jensen LJ, von Mering C, Bork P (2016). eggNOG 4.5: a hierarchical orthology framework with improved functional annotations for eukaryotic, prokaryotic and viral sequences. Nucleic Acids Res.

[45] Moriya Y, Itoh M, Okuda S, Yoshizawa AC, Kanehisa M (2007). KAAS: an automatic genome annotation and pathway reconstruction server. Nucleic Acids Res.

[46] Oliveros JC. Venny. An interactive tool for comparing lists with Venn’s diagrams. 2007-2015. http://bioinfogp.cnb.csic.es/tools/venny/index.html.

