# Supplementary material for: Identification of candidate infection genes from the model entomopathogenic nematode Heterorhabditis bacteriophora
Source: BMC Genomics. 2017 Jan 3;18:8. doi: 10.1186/s12864-016-3468-6 (PMC5209865; doi:10.1186/s12864-016-3468-6)
Supplement: Additional file 1: Table S1. — Summary of Illumina sequencing reads. Figure S1 RNA-seq Analysis Pipeline. Pipeline used to collect, trim and analyze RNA-seq data. RNA sequencing reactions were performed by the Institute of Genome Sciences (University of Maryland School of Medicine) using high quality total RNA obtained from IJs incubated in hemolymph plasma (9 h) or Ringer’s solution (0 h). The resulting reads were trimmed, screened for quality and mapped to the H. bacteriophora reference genome. Expression analysis for DEGs was performed using the edgeR package (PDF 634 kb) [file 12864_2016_3468_MOESM1_ESM.pdf]

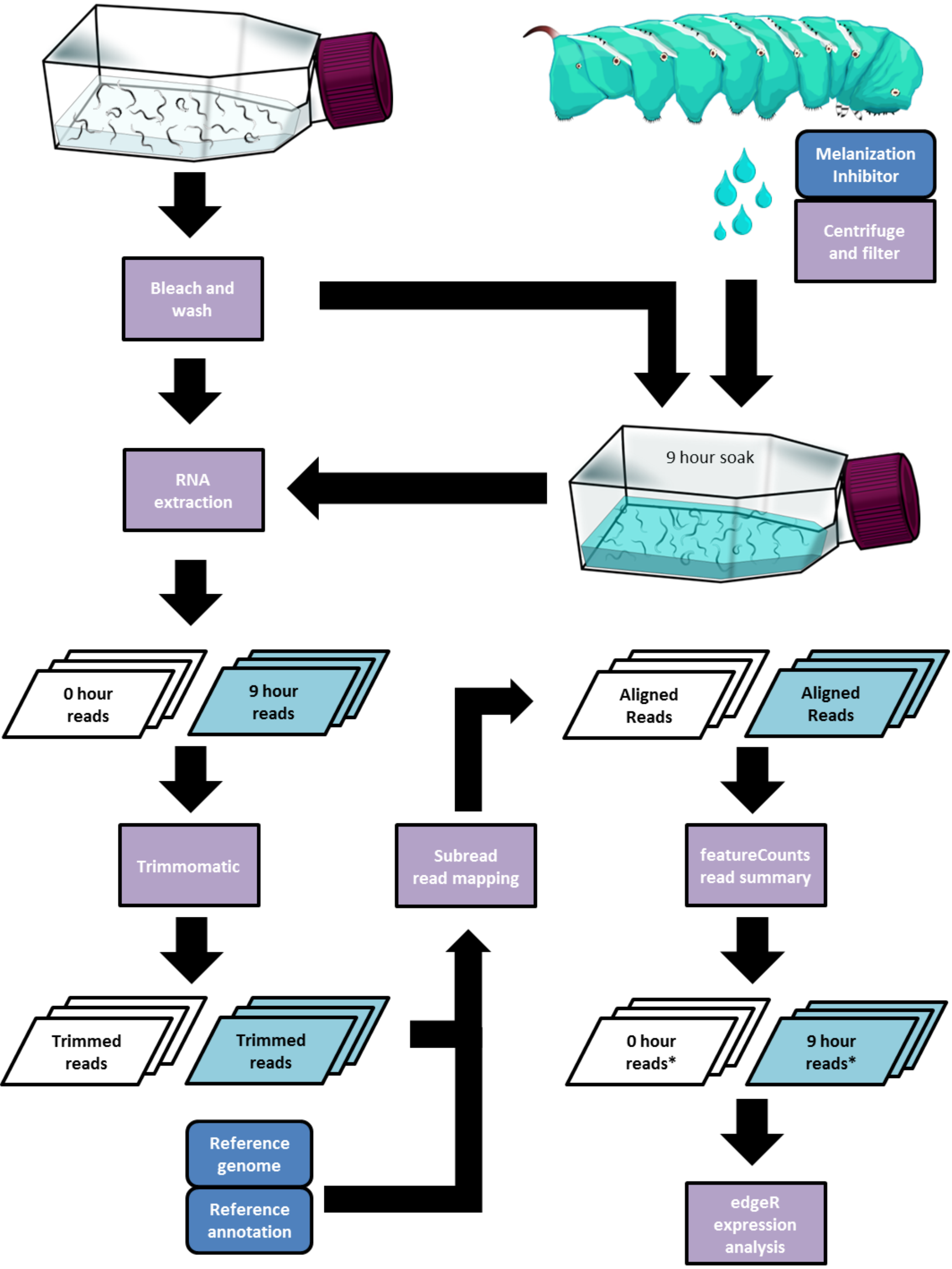
Melanization Inhibitor
Centrifuge and filter
Bleach and wash
9 hour soak
RNA extraction
0 hour reads
9 hour reads
Aligned Reads
Aligned Reads
Trimmomatic
Subread read mapping
featureCounts read summary
Trimmed reads
Trimmed reads
0 hour reads*
9 hour reads*
Reference genome
Reference annotation
edgeR expression analysis

Additional Table 1 Summary of Illumina sequencing reads

Read Status	Control (n = 3)	Hemolymph (n = 3)
Assigned	20115229	21190420
Ambiguous	255192	178649
No Feature	13184325	16030003
Unmapped	4084703	8600584
Total Reads	37639450	45999656
Total Mapped	33554746	37399072
